# Novel Potential Risk Loci for Migraine in the Portuguese Population

**DOI:** 10.3390/ijms27125165

**Published:** 2026-06-06

**Authors:** Rodrigo De Marco, Kevin Pucci, Mariana Santos, Raquel Gil-Gouveia, Bruno Cavadas, Alda Sousa, Miguel Alves-Ferreira, Luísa Azevedo, Carolina Lemos, Andreia Dias

**Affiliations:** 1 Unit for Multidisciplinary Research in Biomedicine (UMIB), School of Medicine and Biomedical Sciences (ICBAS), University of Porto, Rua Jorge Viterbo Ferreira 228, 4050-313 Porto, Portugalcclemos@icbas.up.pt (C.L.); 2 ITR-Laboratory for Integrative and Translational Research in Population Health, Rua das Taipas 135, 4050-600 Porto, Portugal; 3 i3S-Instituto de Investigação e Inovação em Saúde, Universidade do Porto, Rua Alfredo Allen 208, 4200-135 Porto, Portugal; 4 Hospital da Luz Headache Center, Neurology Department, Hospital da Luz Lisboa, Avenida Lusíada 100, 1500-650 Lisbon, Portugal; 5 Center for Interdisciplinary Research in Health, Universidade Católica Portuguesa, Palma de Cima, 1649-023 Lisbon, Portugal; 6 CGPP-Centro de Genética Preditiva e Preventiva, i3S-Instituto de Investigação e Inovação em Saúde, Universidade do Porto, Rua Alfredo Allen 208, 4200-135 Porto, Portugal

**Keywords:** migraine, GWAS, polygenic risk score, synaptic transmission, vascular disease

## Abstract

Common forms of migraine are complex disorders characterized by significant clinical diversity. Their genetic basis has been extensively studied but remains unclear. This study represents the first pilot genome-wide association study (GWAS) integrating a polygenic risk score (PRS) in the Portuguese population, designed to identify migraine susceptibility loci through a case–control study and unravel population-specific variants. Genotyping data was acquired with Applied Biosystems Axiom™ PMDA array, producing 12,035,248 single-nucleotide polymorphisms (SNPs) post-imputation, providing a comprehensive scope for GWAS analysis. PRS models were created and tested using a k-folds cross-validation framework and the optimal significance threshold was assessed. We detected 12 potential risk loci corresponding to 12 lead SNPs (*RP11-204N11.2*, *CTA-481E9.4*/*CTA-481E9.3*, *RAP1A*, *TIGD4*, *CADPS2*, *RP11-46E17.6*, *RP4-569D19.5*, *RP11-398K14.1*, *PCBP1-AS1*, *TCF15*, *IL6R* and *UNC13A*). The top three variants (*RP11-204N11.2*, *CTA-481E9.4*/*CTA-481E9.3* and *RAP1A*) were also supported by the PRS model. We highlight that several variants present putative biological relevance to migraine pathophysiology, reinforcing the importance of neurotransmitter release, synaptic transmission and the involvement of vascular components in migraine pathophysiology.

## 1. Introduction

The most common forms of migraine with and without aura (MwA/MwoA) are complex disorders characterized by significant clinical heterogeneity. Initially attributed solely to dysregulated vascular mechanisms, current evidence on migraine-related pathophysiological processes also underscore the role of neuronal mechanisms, which include cortical spreading depression and synaptic plasticity associated with central sensitization [1,2]. The pathophysiology of these disorders is only partially understood but involves the activation of the trigeminovascular system (TVS), responsible for pain sensation. TVS activation mediates the release of neuropeptides, such as calcitonin gene-related peptide (CGRP), substance P, neurokinin A, vasoactive intestinal peptide (VIP), pituitary adenylate cyclase-activating peptide (PACAP) and nitric oxide [3,4]. Several migraine therapies act on CGRP release, including triptans, ditans, CGRP receptor blockers (gepants) and CGRP-related monoclonal antibodies, considered a breakthrough in migraine-specific treatments [5]. In addition to CGRP, the PACAP pathway might prove to be a useful neuropeptide system for preventing and/or blocking migraine attacks.

Genome-wide association studies (GWAS) in migraine have allowed the identification of several loci that harbor genetic risk factors [6], which have low penetrance individually but together might have a significant impact on disease susceptibility. More recently, a GWAS meta-analysis of migraine reported 123 genomic loci, of which 86 were previously unknown, and included new risk loci containing target genes (*CALCA* and *CALCB*) associated with the CGRP pathway and the serotonin 5-HT1F receptor [7]. Nonetheless, population-specific GWAS remain crucial to understanding intrapopulation genetics’ heterogeneity [8].

Given migraine’s polygenic nature, several statistical methods have gained prominence in recent years, with the polygenic risk score (PRS) standing out as a valuable tool to quantify a given phenotype’s relative risk. It is computed as a linear combination of single-nucleotide polymorphisms (SNPs), where each SNP is weighted by its effect size and then summed (*PRS* = Σ *SNPi* × *βi*, with *SNPi* representing the effect allele count and *βi* the effect size) [9]. By accounting for multiple SNPs simultaneously, it is possible to increase the model’s predictive power [10]. Using GWAS data from 375,000 individuals, a PRS model evaluated response to common acute migraine therapies and highlighted variants that possibly help to explain patient response to triptans, supporting the potential of precision medicine in migraine [11].

Overall, GWAS and PRS can provide novel, clinically valuable insights into population and disease-associated genetic landscapes, enabling therapeutic developments and precision medicine strategies. Describing population-specific susceptibility loci is crucial to unraveling the pathophysiology of migraine, so we present herein the first GWAS in the Portuguese population, aimed at identifying migraine susceptibility risk loci through a case–control study. We have additionally generated a PRS model, allowing a weighted identification of the SNPs most strongly associated with migraine phenotypes.

## 2. Results

### 2.1. Characterization of Sample Study

We analyzed the quality-controlled genotypes of 341 individuals, with a case–control ratio close to 1:1. At the time of observation, the mean age was slightly higher for controls than migraineurs (39.33 ± 13.98 vs. 37.18 ± 11.59 years), increasing confidence that controls are, indeed, migraine-free (Table 1). MwA and MwoA subgroups were similar in size. However, given the limited sample size, no stratification of migraineurs was performed.

### 2.2. Identification and Characterization of Potential Migraine Risk Loci

**Figure 1:**
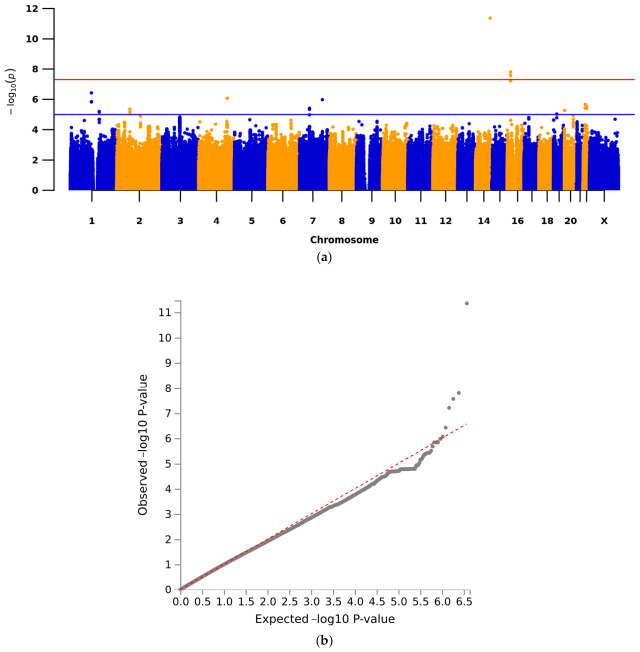
Manhattan plot and quantile–quantile (Q–Q) plot for migraine susceptibility. (**a**) Manhattan plot for migraine risk in the Portuguese population. The red line indicates the *p*-value threshold for genome-wide significance (*p* = 5 × 10^−8^) and the blue line suggestive significance (*p* = 1 × 10^−5^); (**b**) Q–Q plot for migraine susceptibility in the Portuguese population.

A Manhattan plot was generated from the resulting data of 12,035,248 SNPs associated with migraine susceptibility (Figure 1a). The quantile–quantile (Q–Q) plot (Figure 1b) and the genomic inflation factor (lambda, λ = 1.035—indicating that the *p*-values were not inflated) showed no evidence of systematic bias.

We identified 12 potential genomic risk loci—2 within the genome-wide significance threshold (*p*-value < 5 × 10^−8^) and another 10 within the suggestive significance threshold (*p*-value < 1 × 10^−5^)—corresponding to 12 lead SNPs (three intronic, seven intergenic and two ncRNA) (Table 2). Regarding variants in high linkage disequilibrium (LD) (r^2^ > 0.6) with the lead SNPs—henceforth candidate SNPs—a total of 260 variants were found (Supplementary Table S1), of which 122 (46.9%) were located within protein-coding genes, 87 (33.5%) were intergenic, and 47 (18.1%) were ncRNA variants. A total of 4 of the 260 candidate SNPs surpassed FUMA’s suggested deleteriousness threshold, suggesting a potential functional impact [12].

We assessed lead SNPs, as well as several candidate SNPs (*p* < 1 × 10^−5^), for possible regulatory functions using RegulomeDB (RDB) (Table 3). One intergenic locus—containing a lead SNP near *CADPS2* (rs10240812)—likely affects binding of transcription factors and/or other regulatory elements (RDB score = 2b). Additionally, nine other loci seem to have minimal transcription factor (TF) binding evidence (RDB score = 5, for the *CTA-481E9.4*/*CTA-481E9.3*, *RAP1A*, near *TIGD4*, *PCBP1-AS1* and *IL6R* loci, or RDB score = 6, for the near *RP4-569D19.5*, near *RP11-398K14.1* and near *TCF15* loci). Moreover, eight candidate SNPs with suggestive significance show at least minimal binding evidence—this includes variants in *RAP1A* and *IL6R*, as well as a variant in *PCBP1-AS1* with RDB score of 2b.

HaploReg analysis shows eQTL hits for several of the lead and candidate SNPs—namely those of *RAP1A* (and the partially overlapping *FAM212B* gene from the complement strand), near *TIDG4*, *PCBP1-AS1*, near *TCF15* and *IL6R*—which indicates possible impact on gene expression. Detailed information about the potential regulatory impacts of lead and candidate SNPs is shown in Supplementary material, including HaploReg analysis (Supplementary Table S2).

### 2.3. Enrichment Analysis

We identified 42 gene ontology (GO) terms significantly associated with five of the potential risk loci—*RAP1A*, near *CADPS2*, near *TCF15*, *IL6R* and *UNC13A* (Supplementary Table S3). Notably, GO terms results were particularly rich in immune-associated, signal transduction and neurobiological processes, including some related to neural synapse modulation (GO:0016081, GO:2000300, GO:0035249 and GO:0098978). Our top enriched GO term—Positive Regulation of Cell Differentiation (GO:0045597)—is also highlighted in findings from the largest migraine GWAS available, reported by Hautakangas et al. [7]. No statistically significant pathways were identified in either Kyoto Encyclopaedia of Genes and Genomes (KEGG) or Reactome.

### 2.4. Notable Potential Risk Loci of Migraine Susceptibility

**Figure 2:**
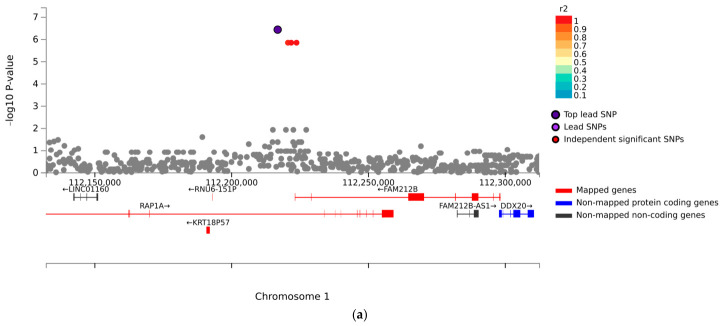

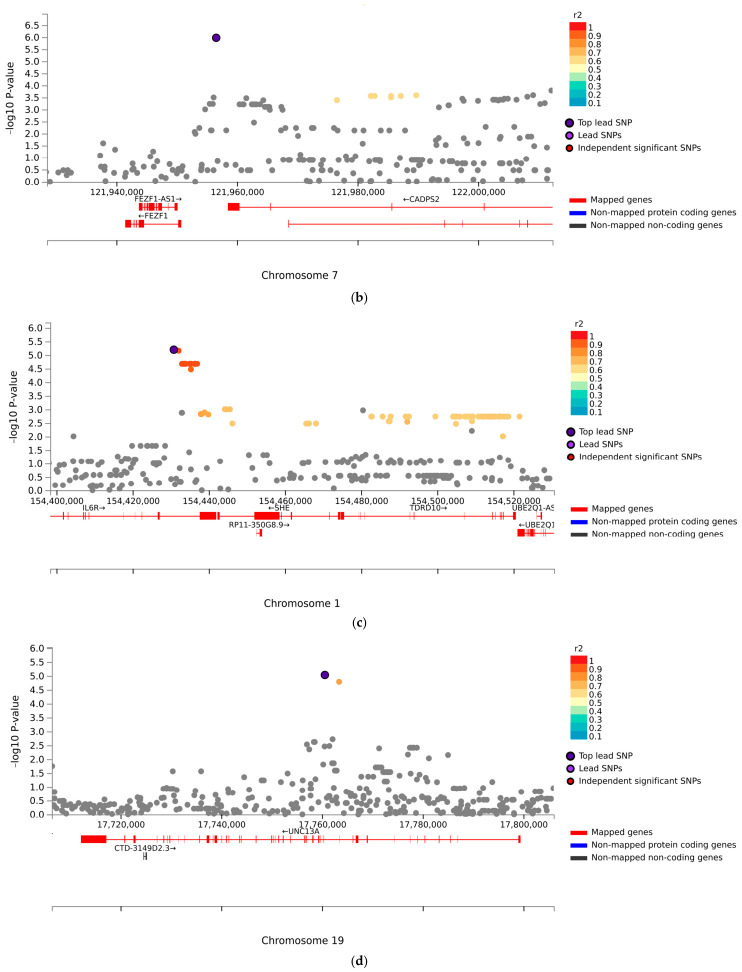
Regional locus plots of four genes potentially associated with migraine pathways. The plots display the genomic regions containing the top lead single nucleotide polymorphisms (SNPs) associated with: (**a**) *RAP1A* [rs7525578], (**b**) *CADPS2* [rs10240812], (**c**) *IL6R* [rs10908839] and (**d**) *UNC13A* [rs10908839]. Lead SNPs, shown as a purple circle, and the remaining SNPs, with colors indicating the level of linkage disequilibrium (r^2^) with the top lead SNP. The x-axis shows the chromosomal location, and the y-axis shows the negative log10 of the genome-wide association study adjusted *p*-value. Genes within each given locus are displayed under the corresponding regional plot.

Four of the new potential risk loci highlight genes (*RAP1A*, near *CADPS2*, *IL6R* and *UNC13A*) that encode proteins related to hallmark processes of migraine pathophisiology, namely neuromodulation, inflammation and synaptic transmission. We observed an association with suggestive significance at chromosome 1 in the *RAP1A* gene (lead SNP rs7525578, *p* = 3.765 × 10^−7^; Figure 2a). We additionally found loci on chromosome 7, 1 and 19, containing the *CADPS2* (lead SNP rs10240812, *p* = 1.057 × 10^−6^; Figure 2b), *IL6R* (lead SNP rs10908839, *p* = 6.331 × 10^−6^; Figure 2c) and *UNC13A* genes (lead SNP rs11665951, *p* = 9.213 × 10^−6^; Figure 2d), respectively.

Enabled by STRING, protein–protein interaction maps were generated and analyzed to assess possible interactions between the four genes’ proteins (RAP1A, CADPS2, IL6R and UNC13A) and proteins associated with known migraine pathways and therapeutic targets. No reliable interactions were identified. Interaction maps for single-molecule queries (each protein of interest) can be found in Supplementary material (Supplementary Figure S1).

### 2.5. Polygenic Risk Score

We report modest but higher-than-chance values of AUC and pseudo-R^2^—approximately 0.71 and 0.17, respectively—with performance increases following decreases of the *p*-value threshold, meaning the best predictive models are, purportedly, the ones with higher stringency (Supplementary Figure S2).

We analyzed the SNPs within suggestive significance (*p* < 1 × 10^−5^) selected in more than 50% of iterations of the k-folds cross-validation process to determine whether any previously reported migraine-associated variants were present. The three selected SNPs (Table 4) did not show reporting in ClinVar nor previous associations with migraine but exactly matched the top three GWAS hits—rs117797734, rs62044126 and rs7525578—and, interestingly, the *RAP1A* variant (rs7525578) is tagged in a GWAS catalog entry related to Moyamoya disease, a rare intracranial arteriopathy disorder which can clinically mimic migraine [13,14].

## 3. Discussion

This study reports, for the first time in the Portuguese population, a pilot GWAS followed up with PRS calculation aimed at uncovering genetic factors that could be involved in migraine susceptibility. GWAS identified a total of 12 loci and, among these, four loci (*RAP1A*, near *CADPS2*, *IL6R* and *UNC13A*) are involved in seemingly biologically relevant pathways to migraine pathophysiology. The putative biological relevance of other identified loci still needs to be further investigated. Regarding the PRS analysis, the models’ performance—assessed by AUC and Nagelkerke’s pseudo-R^2^—was modest but seemingly above-chance, improving under increasing stringency of the *p*-value thresholding. Having selected a reasonable model (*p* = 1 × 10^−5^), it is noteworthy that the selected SNPs exactly match the top three SNPs selected by the GWAS and that none of the variants had any previous indication of pathogenicity in the context of migraine disorders.

It is important to underscore that the limited replication of GWAS across different populations can be attributed to variations in genetic architecture. Ethnically diverse groups exhibit differences due to population-specific variations and changes in allele frequency resulting from genetic drift, local selection, or both. Moreover, founder effects are known to play a role in prevalence variations of complex diseases among populations [8]. Cross-study comparisons indicate potential variations in migraine prevalence based on ethnicities, which could stem from methodological aspects, ethnicity-specific variations in genetic predisposition, environmental risks, or cultural influences affecting symptom reporting [15]. These differences between populations can translate into different clinical manifestations and disparities in therapeutic response. Often, diverse populations are studied as part of large meta-analyses that combine data to estimate associations to identify variants with consistent effects across populations, but this hinders the detection of population-specific genetic risk factors [8].

### 3.1. RAP1A Is Involved in Synapse Plasticity and Inflammation

*RAP1A* encodes a small GTP-binding protein of the Ras superfamily, with roles in cell adhesion and linked to neuronal development, synaptic signaling and immune-cell function. In neurons, RAP1A contributes to neurite outgrowth and participates in Ras/Rap signaling pathways that regulate synaptic plasticity [16,17]. Importantly, RAP1 activation enables PACAP-induced signaling, a key neurotransmitter in migraine pathophysiology, it having been suggested that the anti-CGRP therapeutic success could possibly be reproduced by the use of anti-PACAP monoclonal antibodies [18]. Furthermore, dysregulation of Rap GTPases has been linked to neurodevelopmental disorders, including autism, intellectual disability, and schizophrenia [19]. RAP1A also plays a central role in immunity and inflammation, being involved in the coordination of immune cell recruitment and homeostasis [20]. This draws further interest into its association with Moyamoya disease, an intracranial arteriopathy, given its preponderant neurovascular component—in fact, Moyamoya disease and migraine may present with similar neurological symptoms, requiring imaging studies for differential diagnosis [13,21]. Additionally, the *RAP1A* variant identified in this study (rs7525578, *p* = 3.765 × 10^−7^) has been associated with atrial fibrillation, a disease with neurogenic contribution [22].

Taking into account this gene’s apparent biological relevance, our GWAS results, further supported by the PRS modeling, place *RAP1A* in the spotlight. Besides being the third most significant identified variant in this study and the top variant within a protein-coding gene, its links to neurotransmission, neurodevelopmental disorders and intracranial arteriopathy establish a striking parallel with known migraine associations [23,24,25]. *RAP1A* thus seems to link two inextricable aspects of migraine pathophysiology: neuroplasticity and neurovascular mechanisms.

### 3.2. CADPS2 and UNC13A—Complementary Presynaptic Orchestrators

#### 3.2.1. CADPS2

*CADPS2* encodes a calcium-binding protein which localizes to secretory granules and regulates synaptic vesicle exocytosis. CADPS2 is enriched at presynaptic terminals in the cerebellum, where it associates with vesicular structures distinct from classical synaptic vesicles [26]. CADPS2^+^ vesicles have been shown to be rich in neurotrophin-3 (NT-3) and brain-derived neurotrophic factor (BDNF), and CADPS2 has been implicated in dense-core vesicle trafficking [27].

Overexpression and loss-of-function experiments have implicated CADPS2 as a key regulator of neurotrophin secretion during cerebellar development. In fact, Cadps2-null mice show impaired release of NT-3 and BDNF, as well as widespread abnormalities in cerebellar morphogenesis, synaptic organization and motor learning [27]. The mice also display “autistic-like” phenotypes, including altered social behaviors and changes in cerebellar circuitry [28]. Importantly, human studies have further suggested a link between CADPS2, intelligence, memory and autism spectrum disorder, as well as Alzheimer’s (AD) and Parkinson’s diseases [29,30].

The variant found—rs10240812, intergenic but located less than 2kb downstream of *CADPS2*—might have an impact on gene expression, as suggested by its low RegulomeDB score (RDB score = 2b). Considering the gene’s role in synaptic transmission and neurological disorders, it is not too far-fetched to hypothesize that such a change could affect neuronal circuitry in a way that increases migraine propensity. However, GWAS confidence must first be increased to confirm this exploratory result, as it fell short of genome-wide significance (*p* = 1.057 × 10^−6^), and further studies would then be required to ascertain the functional effects that this specific variant exerts.

#### 3.2.2. UNC13A

The *UNC13A* gene (*Munc13-1* in mice) encodes a presynaptic priming factor that plays an essential role in synaptic vesicle fusion, shaping and maintaining synaptic active zones by enabling the priming and docking of vesicles [31]. UNC13A regulates the transition of synaptic vesicles into a release-ready state by interacting with syntaxin-1 and other SNARE components, a protein complex previously linked to migraine susceptibility in the Portuguese population [31,32,33].

Genome-wide association studies identified UNC13A risk variants that modify disease susceptibility and survival in amyotrophic lateral sclerosis (ALS) [34,35]. More recently, TDP-43 pathology—present in almost all ALS and most frontotemporal dementia (FTD) cases—was shown to cause cryptic exon inclusion in UNC13A, leading to nonsense-mediated decay and reduced protein levels [36,37]. TDP-43-dependent loss of UNC13A impairs synaptic vesicle priming and is proposed to contribute directly to motor neuron vulnerability. This conditional depletion of UNC13A helps to explain why *UNC13A* risk variants are actually quite frequent in the general population, since their deleteriousness is only made apparent in infrequent cases of TDP-43 pathology [38]. Recent evidence also suggests a role of *UNC13A* variants in epileptic encephalopathies [39].

Loss-of-function studies demonstrate that its depletion severely reduces synaptic vesicle priming and transmission [31]. Intriguingly, a homozygous *UNC13A* nonsense mutation has been reported in a single case of a patient with, among other symptoms, cortical hyperexcitability, which closely relates to hypersensitivity and cortical spreading depression—hallmarks of migraine attacks [40,41].

*UNC13A*’s involvement in neurological disease—especially in key migraine processes, namely neurotransmission and cortical hyperexcitability—adds potential biological relevance to the potential risk locus identified in this pilot study (rs11665951, *p* = 9.213 × 10^−6^). Further substantiation of the risk posed by this intriguing variant is warranted.

### 3.3. Additional Noteworthy Variants

The most significant variant in this GWAS (rs117797734, *p* = 4.268 × 10^−12^) is intergenic, located in a relatively “deserted” genomic locus, otherwise devoid of notable SNPs and genes—the nearest gene is a non-mapped non-coding gene, *RP11-204N11.2*, ~29 kb away, and the nearest mapped protein-coding gene is almost 1Mb away. The sparsity of SNPs in LD with this top variant emphasizes a high recombination rate, whereas the considerable distance to the nearest genes may entail a role as a long-distance enhancer. Intriguingly, in a GWAS delving into the risk factors of tumefactive demyelination in multiple sclerosis patients, the same variant was identified with similarly high significance (*p* = 2.06 × 10^−11^) [42]. As is the case with most intergenic variants, drawing conclusions regarding functional effects is rather challenging. Nonetheless, keeping track of other instances of this variant’s identification in large genome-wide studies in neurosciences could help uncover notable patterns.

Another noteworthy lead SNP lies within the *PCBP1-AS1* genomic locus (rs114557033, *p* = 4.38 × 10^−6^). *PCBP1-AS1* is a lncRNA that acts as a competing endogenous RNA (ceRNA)—effectively sponging several miRNAs—and has been implicated in ischemic stroke propagation by increasing proinflammatory factor expression and in inflammatory processes in severe burn lesions [43,44]. Furthermore, it has been found to be particularly enriched within the content of saliva-derived extracellular vesicles, collected from older populations, alongside several transcripts associated with neurodegeneration [45]. Considering the importance of meningeal inflammation in migraine nociception, it may be worth investigating further whether variants in *PCBP1-AS1* could have potentiating effects in migraine attacks.

Finally, we identified a variant within the *IL6R* genomic locus (rs10908839, *p* = 6.331 × 10^−6^), a gene encoding a receptor subunit for the well-known IL6 proinflammatory cytokine. Interestingly, a soluble isoform of IL6R, when complexed with its ligand, can perform “trans-signaling” via gp130, which expands its target cell pool and has been linked to chronic inflammation [46]. IL6/IL6R have been implicated in several pathologies over time, including some classically neuroinflammation-driven diseases, such as AD and neuromyelitis optica spectrum disorder (NMOSD), as well as myasthenia gravis (MG) [47,48]. In fact, IL6R has been shown to be upregulated in neuroinflammatory-type astrocytes of AD patients, and it is a therapeutic target in NMOSD and MG clinical trials, which have already surpassed phase III [47,48]. Our identification of this variant could potentially point to dysregulation of the proinflammatory landscape as a factor increasing migraine risk.

### 3.4. Limitations

As a pilot endeavor, our GWAS is considerably limited by its small sample size—variant significance is mostly suggestive, effect sizes are likely inflated and, consequently, the PRS model may be considerably overfitted [49,50]. Moreover, it builds upon a cohort of patients that stem from a single tertiary hospital, receiving referrals of complex cases, and thus preventing generalizations into community settings. Furthermore, our data are population-specific and thus findings cannot be extrapolated and applied to the global population. Thus, further genotyping data is warranted to improve the statistical power of the analysis and applicability of the PRS framework.

According to established guidelines [50], “if the GWAS data are relatively underpowered, the optimal threshold is more likely to be a p-value of 1”. In our case, the situation is reversed—the model naturally tends to focus on the most relevant variants, shifting the optimal *p*-value threshold closer to 0. Nevertheless, the risk of overfitting must be carefully considered; relevant SNPs may be overlooked in favor of those showing stronger but potentially spurious statistical signals, thereby reducing the model’s accuracy and generalizability. Conversely, applying overly stringent *p*-value thresholds may inadvertently exclude variants that are genuinely associated with the phenotype or which contribute to the pathophysiology of migraine [50]. Importantly, as an extension of the GWAS it builds upon, the PRS is also exploratory.

It is also important to emphasize that a statistical association between a phenotype and an SNP does not necessarily indicate a causal relationship. A non-functional SNP may appear associated with the phenotype due to strong linkage disequilibrium with the true causal variant. If the biologically meaningful SNP is removed during quality control—owing to low MAF, Hardy-Weinberg disequilibrium, or missing data—a proxy SNP may be retained as the regional representative. Such substitutions can obscure the true genetic signal and complicate the biological interpretation of the results [51].

## 4. Materials and Methods

### 4.1. Subjects and Study Design

This case–control study was conducted in a Portuguese cohort from the outpatient neurology clinic at Centro Hospitalar Universitário de Santo António (CHUdSA) in coordination with Centre for Predictive and Preventive Genetics—Institute for Research and Innovation in Health (CGPP-i3S). A total of 380 samples were genotyped, 28 having been excluded due to inconsistencies regarding age-related data. We thus started analysis with 352 individuals: 172 migraine patients and 180 controls. Clinical information of subjects was collected, and patients with familial hemiplegic migraine were excluded. Controls and cases were of the same ethnic and geographical origin, age-matched and non-related. All cases and controls underwent a diagnostic interview, using the same structured questionnaire, based on the operational criteria of the International Headache Society (IHS)—3rd edition of the International Classification of Headache Disorders (ICHD-3) [52]. Blood samples were collected in the sequence of a neurology appointment and were stored at CGPP’s biobank at i3S. The Ethics Committees of CHUdSA and i3S approved the study and participants gave their written informed consent.

### 4.2. DNA Extraction and Genome-Wide Array Genotyping

Genomic DNA extraction from peripheral blood samples was performed by the standard salting-out method using the QIAamp^®^ DNA Blood Mini Kit (QIAGEN, Venlo, The Netherlands). DNA quantification was performed using NanoDrop™ One (Thermo Fisher Scientific, Waltham, MA, USA). Genotyping of over 900,000 SNPs (900 K) was attained with the Axiom™ Precision Medicine Diversity Array (PMDA, Affymetrix) (Thermo Fisher Scientific, Waltham, MA, USA) and the GeneTitan Multi-Channel (MC) Instrument (Thermo Fisher Scientific, Waltham, MA, USA). Genotyping raw data were analyzed with the Axiom™ Analysis Suite version 5.1 (Thermo Fisher Scientific, Waltham, MA, USA), using the Best Practices Workflow with default settings.

### 4.3. Imputation and Quality Control

Genome-wide data were imputed using the Haplotype Reference Consortium (HRC) panel (r1.1) on the Michigan Imputation Server 2 (https://imputationserver.sph.umich.edu/, accessed on 1 June 2026), applying Eagle v2.4 pre-phasing method and Minimac4 for imputation.

For quality control (QC), the genotyped data of 352 samples was filtered for imputation score > 0.8, minor allele frequency (MAF) > 0.01, sample call rate > 0.95, marker call rate > 0.95 and Hardy-Weinberg equilibrium (HWE) *p*-value > 1 × 10^−6^. Principal component analysis (PCA) and a heterozygosity check were performed to assess the samples’ genetic background, four samples having been signaled as outliers (more than three standard deviations from the mean) and excluded from analysis (Supplementary Figure S3). Seven additional samples were excluded due to mismatching stated and biological sex data.

After imputation and QC, a total of 341 samples (167 cases and 174 controls) and 12,035,248 SNPs proceeded to analysis.

### 4.4. GWAS and Statistical Analysis

The Manhattan plot was generated using the qqman v0.1.9 R package. Quantile–quantile (Q–Q) and regional locus plots were generated using the FUMA v1.8.3 software. The significance threshold for genotyped variants was set at *p* = 1 × 10^−5^ (suggestive significance).

### 4.5. Identification of Lead and Candidate SNPs Associated with Migraine

Association analysis for each SNP with migraine susceptibility was performed using PLINK v1.9.0-b.7.11, based on a logistic regression test for each SNP to the phenotype of interest. Sex, age and the first three principal components were used as logistic regression covariates.

Independent significant SNPs (r^2^ < 0.6) were filtered with FUMA software with *p*-values below Bonferroni-corrected suggestive significance (two-tailed *p* < 1 × 10^−5^). All SNPs in LD (r^2^ ≥ 0.6) to one of the independent significant SNPs were defined as candidate SNPs. All independent significant SNPs with r^2^ < 0.1 were defined as lead SNPs and merged into one locus if located within 250kb of one another. LD analyses were conducted using the 1000G Phase3 EUR reference panel.

### 4.6. Gene Mapping

Functional annotations of SNPs were obtained from FUMA: CADD (12.37 as the score threshold for deleteriousness), RegulomeDB and 15-core chromatin states [12]. Additionally, significant expression quantitative trait loci (eQTL) values (false discovery rate (FDR) < 0.05), according to GTEx V8, were selected to map SNPs to genes.

Regulatory genomic data (such as enhancers and TF binding sites) were analyzed to interpret the potential impact of non-coding variants. FUMA results were complemented by HaploReg v4.2 (r^2^ > 0.8) analysis, exploring variants in LD with lead SNPs.

### 4.7. Enrichment Analysis

To identify known biological pathways and gene sets associated with the proposed risk loci, enrichment analysis was performed via Enrichr (https://maayanlab.cloud/Enrichr/, accessed on 1 June 2026) with GO, KEGG and Reactome databases.

### 4.8. Protein Interactions

STRING (v12.0) was used to search for protein–protein interactions (PPI), including direct (physical) and indirect (functional) associations (minimum required interaction score set at 0.400).

### 4.9. Polygenic Risk Score

For QC and PRS calculation, we used the PLINK software v1.9.0-b.7.11, as previously described [50]. QC was performed according to established PLINK guidelines, ensuring that no duplicated SNPs were present in the dataset. Ambiguous SNPs were not an issue due to identical generation conditions. Samples were split into several train/test sets for k-fold cross-validation, ensuring that overlapping samples were also not an issue. Similarly to the standard GWAS, SNPs were excluded based on MAF > 0.01, sample call rate > 0.95, marker call rate > 0.95 and HWE *p*-value > 1 × 10^−6^.

PRS was calculated using k-fold cross-validation (k = 5) of a clumping and thresholding method implemented in PLINK. To account for LD, clumping was performed with the following parameters: *p*-value ≤ 1 (including all SNPs), r^2^ ≥ 0.1, and clump SNPs with distance < 250 kb. Thresholding was then applied to exclude SNPs with high *p*-values, reducing noise and potential overfitting. Following the recommended guidelines [50], PRS were computed across multiple significance thresholds (1 × 10^−5^, 1 × 10^−4^, 2 × 10^−4^, 5 × 10^−4^, 1 × 10^−3^, 5 × 10^−2^, 0.1, 0.2, 0.3, 0.4, 0.5 and 1) to identify the optimal predictive model, as the best threshold is not known a priori. Model performance was evaluated using mean AUC and Nagelkerke’s pseudo-R^2^, and results were visualized by plotting scores against *p*-value thresholds and comparing PRS distributions between cases and controls. Visualization of PRS results was performed using RStudio version 4.5.3 (11 March 2026 ucrt).

## 5. Conclusions

We report herein the first pilot migraine GWAS in the Portuguese population, detecting 12 putative risk loci for migraine susceptibility. We highlight particularly interesting intronic *RAP1A* and *UNC13A* variants, as well as an intergenic variant near the *CADPS2* gene, with potential biological relevance to migraine pathophysiology. Additional variants of interest lie within introns of the *PCBP1-AS1* and *IL6R* genes, as well as a rather isolated intergenic SNP. The findings reinforce the importance of synaptic transmission, as well as the involvement of the vascular component in migraine pathophysiology.

## Figures and Tables

**Table 1:** Demographic and clinical data of migraine patients and controls.

	Migraineurs (n = 167)		Controls (n = 174)
	MwoA (n = 80)	MwA (n = 87)	
Female (n)	73	71	91
Male (n)	7	16	83
Age in years (mean ± SD)	35.75 ± 10.40	38.49 ± 12.51	39.33 ± 13.98
	37.18 ± 11.59		

MwoA—migraine without aura; MwA—migraine with aura; SD—standard deviation.

**Table 2:** Characterization of the 12 genomic loci of interest (potential risk loci).

Locus	SNP	CHR	Position (GRCh37)	A1	A2	MAF	*p*-Value	OR
near *RP11-204N11.2*	rs117797734	14	98284326	T	C	0.01193	4.268 × 10^−12^	7.404 [4.202 13.05]
*CTA-481E9.4* *CTA-481E9.3*	rs62044126	16	18170199	A	C	0.1998	1.575 × 10^−8^	3.437 [2.240 5.273]
*RAP1A*	rs7525578	1	112216961	T	C	0.07853	3.765 × 10^−7^	3.463 [2.144 5.591]
near *TIGD4*	rs6826890	4	153647589	G	C	0.341	8.54 × 10^−7^	2.628 [1.789 3.862]
near *CADPS2*	rs10240812	7	121956540	C	G	0.3638	1.057 × 10^−6^	0.3733 [0.2513 0.5545]
near *RP11-46E17.6*	rs11913002	22	27886736	A	G	0.4165	2.11 × 10^−6^	2.388 [1.666 3.421]
near *RP4-569D19.5*	rs28571767	22	35866387	T	C	0.2773	3.109 × 10^−6^	2.654 [1.761 4.000]
near *RP11-398K14.1*	rs10224317	7	52748202	A	G	0.3976	3.847 × 10^−6^	2.401 [1.656 3.482]
*PCBP1-AS1*	rs114557033	2	70205075	A	G	0.04473	4.38 × 10^−6^	6.424 [2.904 14.21]
near *TCF15*	rs1964858	20	600260	A	G	0.4006	5.381 × 10^−6^	0.4192 [0.2882 0.6097]
*IL6R*	rs10908839	1	154430798	C	G	0.2664	6.331 × 10^−6^	2.616 [1.723 3.971]
*UNC13A*	rs11665951	19	17760561	C	T	0.1451	9.213 × 10^−6^	0.3417 [0.2126 0.5492]

Reference alleles underlined. SNP—single nucleotide polymorphism; CHR—chromosome; A1—effect allele; A2—non-effect allele; MAF—minor allele frequency; OR—odds ratio with 95% confidence interval.

**Table 3:** Annotation of potential regulatory lead SNPs in RegulomeDB categories.

Locus	SNP	RDB Score	Description
near *RP11-204N11.2*	rs117797734	7	No evidence
*CTA-481E9.4* *CTA-481E9.3*	rs62044126	5	TF binding or DNase peak
*RAP1A*	rs7525578	5	TF binding or DNase peak
near *TIGD4*	rs6826890	5	TF binding or DNase peak
near *CADPS2*	rs10240812	2b	TF binding + any motif + DNase footprint + DNase peak
near *RP11-46E17.6*	rs11913002	7	No evidence
near *RP4-569D19.5*	rs28571767	6	Motif hit
near *RP11-398K14.1*	rs10224317	6	Motif hit
*PCBP1-AS1*	rs114557033	5	TF binding or DNase peak
near *TCF15*	rs1964858	6	Motif hit
*IL6R*	rs10908839	5	TF binding or DNase peak
*UNC13A*	rs11665951	5	TF binding or DNase peak

SNP—single nucleotide polymorphism; RDB—RegulomeDB; TF—transcription factor.

**Table 4:** Top PRS SNPs within the suggestive significance threshold (*p* < 1 × 10^−5^).

SNP	P	OR	Folds (%)	EFO ID (GWAS Catalog)	Functional Consequence
rs117797734	1.42 × 10^−9^	7.257 [3.857 13.65]	80	N/A	Intergenic variant
rs62044126	1.00 × 10^−6^	3.325 [2.069 5.343]	80	N/A	*CTA-481E9.4*/*CTA-481E9.3* ncRNA intronic variant
rs7525578	4.05 × 10^−6^	3.658 [2.127 6.293]	80	MONDO_0016820	*RAP1A* intronic variant

SNP—single nucleotide polymorphism; P—average *p*-value across the k-folds; OR—average odds ratio across the k-folds with 95% confidence interval; Folds—percentage of k-folds where SNP significance was below the *p*-value threshold; EFO—Experimental Factor Ontology; N/A—not available.

## Data Availability

The original contributions presented in this study are included in the article/Supplementary Materials. GWAS summary statistics are available at GWAS Catalog (EMBL-EBI) under accession number GCST90838733. Further inquiries can be directed to the corresponding author.

## Supplementary Materials

The following supporting information can be downloaded at: https://www.mdpi.com/article/10.3390/ijms27125165/s1, Supplementary Figure S1: Protein interaction map for RAP1A, CADPS2, IL6R and UNC13A.; Supplementary Figure S2: (a) AUC and (b) Nagelkerke’s pseudo-R^2^ value plotted relative to the *p*-value thresholds set for the PRS model.; Supplementary Figure S3: Principal component analysis (PCA) sample plot between principal component 1 (PC1) and 2 (PC2). Outlier samples, identified by a deviation from mean greater than three standard deviations, are highlighted in red; Supplementary Table S1: 260 candidate SNPs (GWAS; *p* < 1 × 10^−5^) in high LD (r^2^ ≥ 0.6); Supplementary Table S2: In silico predictions (including transcription factor binding sites and expression quantitative trait locus (eQTL)) obtained by HaploReg platform; Supplementary Table S3: gene ontology terms identified by Enrichr. Statistically significant results (adjusted *p*-value < 0.05) highlighted in green.

## Abbreviations

The following abbreviations are used in this manuscript:

AD	Alzheimer’s disease
AUC	Area under curve
CGRP	Calcitonin gene-related peptide
EFO	Experimental Factor Ontology
eQTL	Expression quantitative trait loci
GO	Gene ontology
GWAS	Genome-wide association study
HWE	Hardy-Weinberg equilibrium
KEGG	Kyoto Encyclopaedia of Genes and Genomes
LD	Linkage disequilibrium
MAF	Minor allele frequency
MwA	Migraine with aura
MwoA	Migraine without aura
OR	Odds ratio
PACAP	Pituitary adenylate cyclase-activating peptide
PCA	Principal component analysis
PRS	Polygenic risk score
QC	Quality control
Q–Q	Quantile–quantile
RDB	RegulomeDB
SNP	Single nucleotide polymorphism
TF	Transcription factor
TVS	Trigeminovascular system

## References

[1] Allan W. (1928). The inheritance of migraine. Arch. Intern. Med..

[2] Fila M., Derwich M., Pawlowska E., Blasiak J. (2025). Neural Plasticity in Migraine Chronification. Eur. J. Neurosci..

[3] Dias A., Mariz T., Sousa A., Lemos C., Alves-Ferreira M. (2022). A review of migraine genetics: Gathering genomic and transcriptomic factors. Hum. Genet..

[4] May A., Goadsby P.J. (2001). Substance P receptor antagonists in the therapy of migraine. Expert Opin. Investig. Drugs.

[5] Puledda F., Silva E.M., Suwanlaong K., Goadsby P.J. (2023). Migraine: From pathophysiology to treatment. J. Neurol..

[6] Van Den Maagdenberg A.M.J.M., Nyholt D.R., Anttila V. (2019). Novel hypotheses emerging from GWAS in migraine?. J. Headache Pain.

[7] Hautakangas H., Winsvold B.S., Ruotsalainen S.E., Bjornsdottir G., Harder A.V.E., Kogelman L.J.A., Thomas L.F., Noordam R., Benner C., Gormley P. (2022). Genome-wide analysis of 102,084 migraine cases identifies 123 risk loci and subtype-specific risk alleles. Nat. Genet..

[8] Sirugo G., Williams S.M., Tishkoff S.A. (2019). The Missing Diversity in Human Genetic Studies. Cell.

[9] Dudbridge F. (2013). Power and Predictive Accuracy of Polygenic Risk Scores. PLoS Genet..

[10] Grangeon L., Lange K.S., Waliszewska-Prosół M., Onan D., Marschollek K., Wiels W., Mikulenka P., Farham F., Gollion C., Ducros A. (2023). Genetics of migraine: Where are we now?. J. Headache Pain.

[11] Kogelman L.J.A., Esserlind A.-L., Francke Christensen A., Awasthi S., Ripke S., Ingason A., Davidsson O.B., Erikstrup C., Hjalgrim H., Ullum H. (2019). Migraine polygenic risk score associates with efficacy of migraine-specific drugs. Neurol. Genet..

[12] Watanabe K., Taskesen E., Van Bochoven A., Posthuma D. (2017). Functional mapping and annotation of genetic associations with FUMA. Nat. Commun..

[13] Rifino N., Aamodt A.H., Wiedmann M., Kramer M., Becker J., Guey S., Acerbi F., Herve D., Bersano A. (2025). The Spectrum of Headaches in Moyamoya Angiopathy: From Mechanisms to Management Strategies—A Consensus Review From the NEUROVASC Working Group. Eur. J. Neurol..

[14] GWAS Catalog. Variant: rs7525578. https://www.ebi.ac.uk/gwas/variants/rs7525578.

[15] Stewart W.F., Lipton R.B., Simon D. (1996). Work-related disability: Results from the American migraine study. Cephalalgia.

[16] Zhang L., Zhang P., Wang G., Zhang H., Zhang Y., Yu Y., Zhang M., Xiao J., Crespo P., Hell J.W. (2018). Ras and Rap Signal Bidirectional Synaptic Plasticity via Distinct Subcellular Microdomains. Neuron.

[17] Cherra S.J., Lamb R. (2024). Interactions between Ras and Rap signaling pathways during neurodevelopment in health and disease. Front. Mol. Neurosci..

[18] Guo S., Jansen-Olesen I., Olesen J., Christensen S.L. (2023). Role of PACAP in migraine: An alternative to CGRP?. Neurobiol. Dis..

[19] Stornetta R.L., Zhu J.J. (2011). Ras and Rap signaling in synaptic plasticity and mental disorders. Neuroscientist.

[20] Ahmed S.M., Daulat A.M., Meunier A., Angers S. (2010). G protein βγ subunits regulate cell adhesion through Rap1a and its effector Radil. J. Biol. Chem..

[21] Jeon J.P., Hong E.P., Ha E.J., Kim B.J., Youn D.H., Lee S., Lee H.C., Kim K.M., Lee S.H., Cho W.-S. (2023). Genome-wide association study identifies novel susceptibilities to adult moyamoya disease. J. Hum. Genet..

[22] Lee G.W., Chen J.J., Wang C.H., Chang S.N., Chiu F.C., Huang P.S., Chua S.K., Chuang E.Y., Tsai C.T. (2025). Identification of a new genetic locus associated with atrial fibrillation in the Taiwanese population by genome-wide and transcriptome-wide association studies. Europace.

[23] Mohammad S., Bussu G., Rukh G., Schiöth H.B., Mwinyi J. (2025). Migraine and its major subtypes—With and without aura are associated with polygenic scores for autism. Cephalalgia.

[24] Bahrami S., Hindley G., Winsvold B.S., O’Connell K.S., Frei O., Shadrin A., Cheng W., Bettella F., Rødevand L., Odegaard K.J. (2022). Dissecting the shared genetic basis of migraine and mental disorders using novel statistical tools. Brain.

[25] Malik R., Winsvold B., Auffenberg E., Dichgans M., Freilinger T. (2016). The migraine-stroke connection: A genetic perspective. Cephalalgia.

[26] Sadakata T., Mizoguchi A., Sato Y., Katoh-Semba R., Fukuda M., Mikoshiba K., Furuichi T. (2004). The Secretory Granule-Associated Protein CAPS2 Regulates Neurotrophin Release and Cell Survival. J. Neurosci..

[27] Sadakata T., Kakegawa W., Mizoguchi A., Washida M., Katoh-Semba R., Shutoh F., Okamoto T., Nakashima H., Kimura K., Tanaka M. (2007). Impaired cerebellar development and function in mice lacking CAPS2, a protein involved in neurotrophin release. J. Neurosci..

[28] Sadakata T., Washida M., Iwayama Y., Shoji S., Sato Y., Ohkura T., Katoh-Semba R., Nakajima M., Sekine Y., Tanaka M. (2007). Autistic-like phenotypes in Cadps2-knockout mice and aberrant CADPS2 splicing in autistic patients. J. Clin. Investig..

[29] Bonora E., Graziano C., Minopoli F., Bacchelli E., Magini P., Diquigiovanni C., Lomartire S., Bianco F., Vargiolu M., Parchi P. (2014). Maternally inherited genetic variants of CADPS2 are present in Autism Spectrum Disorders and Intellectual Disability patients. EMBO Mol. Med..

[30] Obergasteiger J., Überbacher C., Pramstaller P.P., Hicks A.A., Corti C., Volta M. (2017). CADPS2 gene expression is oppositely regulated by LRRK2 and alpha-synuclein. Biochem. Biophys. Res. Commun..

[31] Varoqueaux F., Sigler A., Rhee J.S., Brose N., Enk C., Reim K., Rosenmund C. (2002). Total arrest of spontaneous and evoked synaptic transmission but normal synaptogenesis in the absence of Munc13-mediated vesicle priming. Proc. Natl. Acad. Sci. USA.

[32] Lemos C., Pereira-Monteiro J., Mendonça D., Ramos E.M., Barros J., Sequeiros J., Alonso I., Sousa A. (2010). Evidence of syntaxin 1A involvement in migraine susceptibility: A Portuguese study. Arch. Neurol..

[33] Felício D., Dias A., Martins S., Carvalho E., Lopes A.M., Pinto N., Lemos C., Santos M., Alves-Ferreira M. (2023). Non-coding variants in VAMP2 and SNAP25 affect gene expression: Potential implications in migraine susceptibility. J. Headache Pain.

[34] Van Rheenen W., Shatunov A., Dekker A.M., McLaughlin R.L., Diekstra F.P., Pulit S.L., Van Der Spek R.A.A., Võsa U., De Jong S., Robinson M.R. (2016). Genome-wide association analyses identify new risk variants and the genetic architecture of amyotrophic lateral sclerosis. Nat. Genet..

[35] Diekstra F.P., van Vught P.W.J., van Rheenen W., Koppers M., Pasterkamp R.J., van Es M.A., Schelhaas H.J., de Visser M., Robberecht W., Van Damme P. (2012). UNC13A is a modifier of survival in amyotrophic lateral sclerosis. Neurobiol. Aging.

[36] Brown A.L., Wilkins O.G., Keuss M.J., Hill S.E., Zanovello M., Lee W.C., Bampton A., Lee F.C.Y., Masino L., Qi Y.A. (2022). TDP-43 loss and ALS-risk SNPs drive mis-splicing and depletion of UNC13A. Nature.

[37] Ma X.R., Prudencio M., Koike Y., Vatsavayai S.C., Kim G., Harbinski F., Briner A., Rodriguez C.M., Guo C., Akiyama T. (2022). TDP-43 represses cryptic exon inclusion in the FTD–ALS gene UNC13A. Nature.

[38] Willemse S.W., Harley P., Van Eijk R.P.A., Demaegd K.C., Zelina P., Pasterkamp R.J., Van Damme P., Ingre C., Van Rheenen W., Veldink J.H. (2023). UNC13A in amyotrophic lateral sclerosis: From genetic association to therapeutic target. J. Neurol. Neurosurg. Psychiatry.

[39] Su K., Ma Y., Zhou M., Liu Y., Li C., Jiang Y., Wu Q., Peng G., Wang Y., Fan S. (2025). De novo missense variants of UNC13A are implicated in epileptic encephalopathies and neurodevelopmental disorders. Genes Dis..

[40] Engel A.G., Selcen D., Shen X.M., Milone M., Harper C.M. (2016). Loss of MUNC13-1 function causes microcephaly, cortical hyperexcitability, and fatal myasthenia. Neurol. Genet..

[41] Gollion C. (2021). Cortical excitability in migraine: Contributions of magnetic resonance imaging. Rev. Neurol..

[42] Zhao-Fleming H.H., Decker P.A., Kosel M.L., Drucker K.L., Kollmeyer T., Lachance D.H., Clarkson B.D., Howe C.L., Jenkins R., Tobin W.O. (2025). Genomewide association study of a homogeneous multiple sclerosis cohort: Tumefactive demyelination. Mult. Scler. J..

[43] He Z., Chen L., Zhang W. (2023). LncRNA PCBP1-AS1 Induces Cerebral Ischemia/Reperfusion Injury via the miR-506-3p/CCL2 Axis. Ann. Clin. Lab. Sci..

[44] Tang G., Zhang T., Wang X., Song Z., Liu F., Zhang Q., Huo R. (2018). Sub-pathway analysis for severe burns injury patients: Identification of potential key lncRNAs by analyzing lncRNA-mRNA profile. Exp. Ther. Med..

[45] Wen S., Yu C., Kelsey M.M.G., Pereira M., Alaimo H., Teixeira E., Pracht J., Daiello L.A., Drake J., Sedivy J.M. (2026). RNA transcripts in salivary extracellular vesicle cargo isolated from aged populations. Front. Aging.

[46] Garbers C., Thaiss W., Jones G.W., Waetzig G.H., Lorenzen I., Guilhot F., Lissilaa R., Ferlin W.G., Grötzinger J., Jones S.A. (2011). Inhibition of classic signaling is a novel function of soluble glycoprotein 130 (sgp130), which is controlled by the ratio of interleukin 6 and soluble interleukin 6 receptor. J. Biol. Chem..

[47] Siciliano B., Henkel N.D., Ryan V.W.G., Imami A.S., Vergis J.M., Xu C., Arvay T.O., Sahay S., Pulvender P., Hamoud A.R. (2025). Proinflammatory transcriptomic and kinomic alterations in astrocytes derived from patients with familial Alzheimer’s disease. Brain Behav. Immun. Health.

[48] Li X., Zhao C. (2025). Interleukin-6 in neuroimmunological disorders: Pathophysiology and therapeutic advances with satralizumab. Autoimmun. Rev..

[49] Xiao R., Boehnke M. (2009). Quantifying and correcting for the winner’s curse in genetic association studies. Genet. Epidemiol..

[50] Choi S.W., Mak T.S.-H., O’Reilly P.F. (2020). Tutorial: A guide to performing polygenic risk score analyses. Nat. Protoc..

[51] Dickson S.P., Wang K., Krantz I., Hakonarson H., Goldstein D.B. (2010). Rare Variants Create Synthetic Genome-Wide Associations. PLoS Biol..

[52] Headache Classification Committee of the International Headache Society (IHS) (2018). The International Classification of Headache Disorders, 3rd edition. Cephalalgia.

