# Supplementary figures and images for: Novel Potential Risk Loci for Migraine in the Portuguese Population
Source: Int J Mol Sci. 2026 Jun 6;27(12):5165. doi: 10.3390/ijms27125165 (PMC13300717; doi:10.3390/ijms27125165)

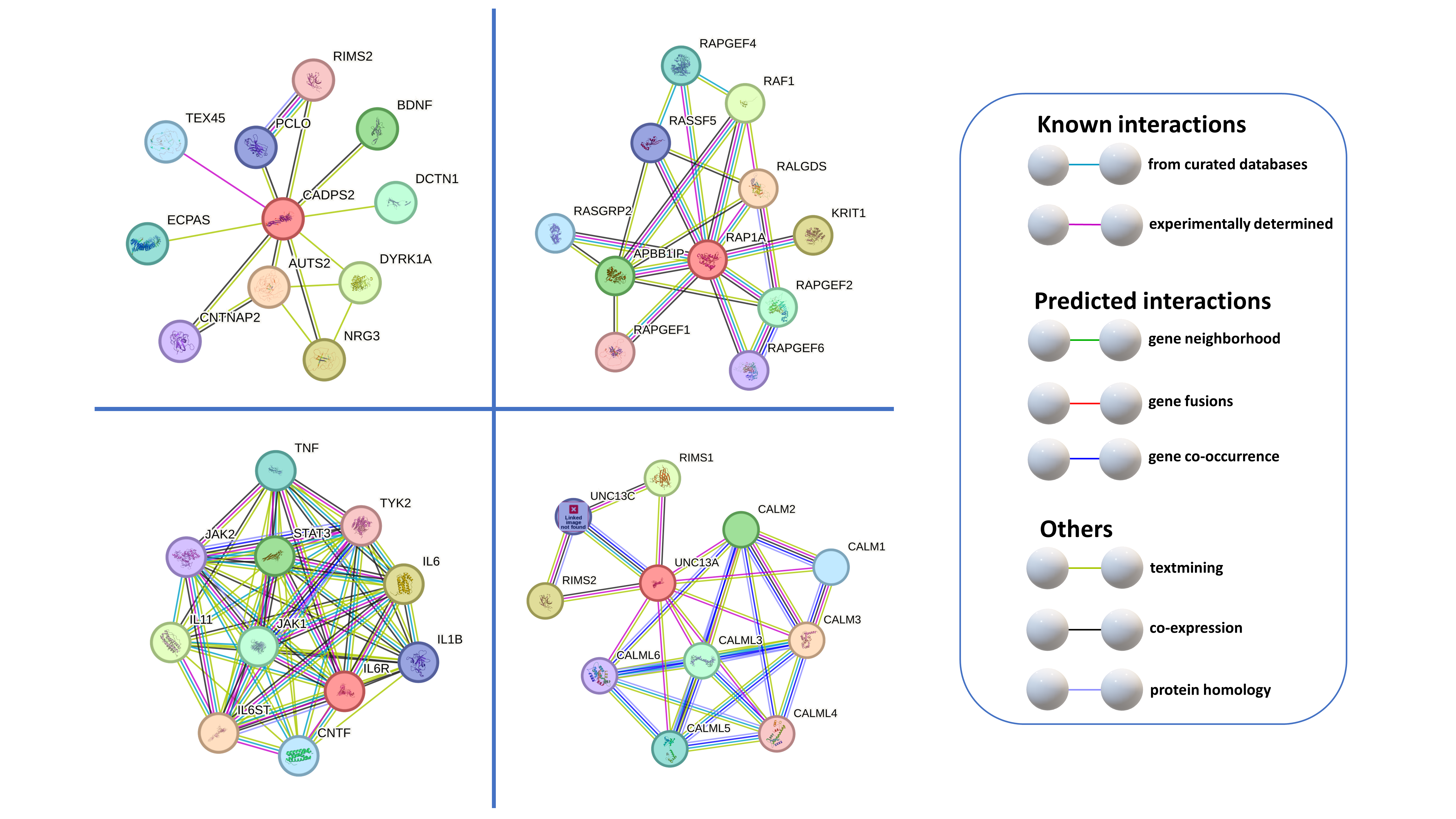

Supplement: Supplementary file 1 [file ijms-27-05165-s001.zip › Supplementary Figure S1.TIF]

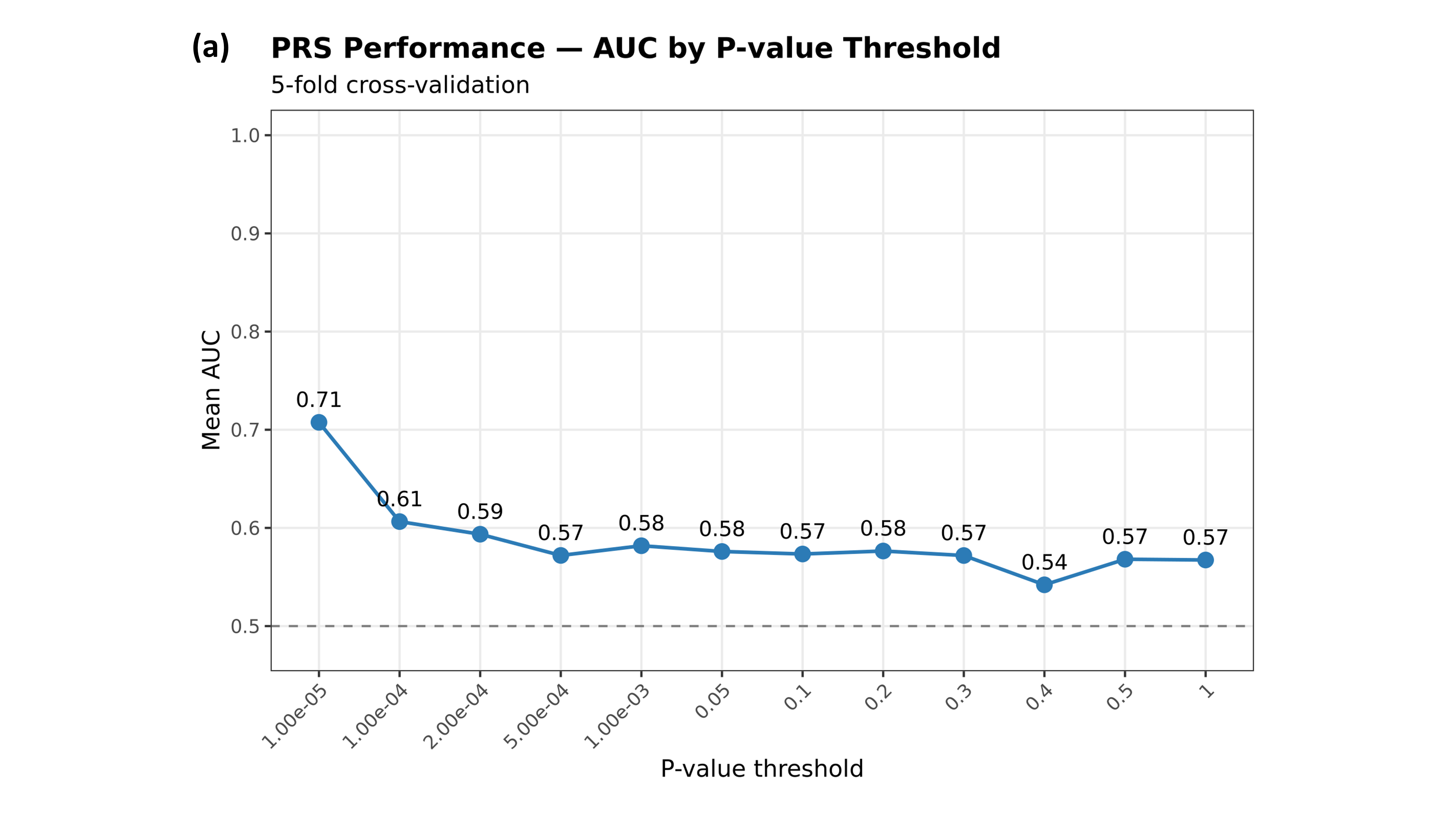

Supplement: Supplementary file 1 [file ijms-27-05165-s001.zip › Supplementary Figure S2a.tif]

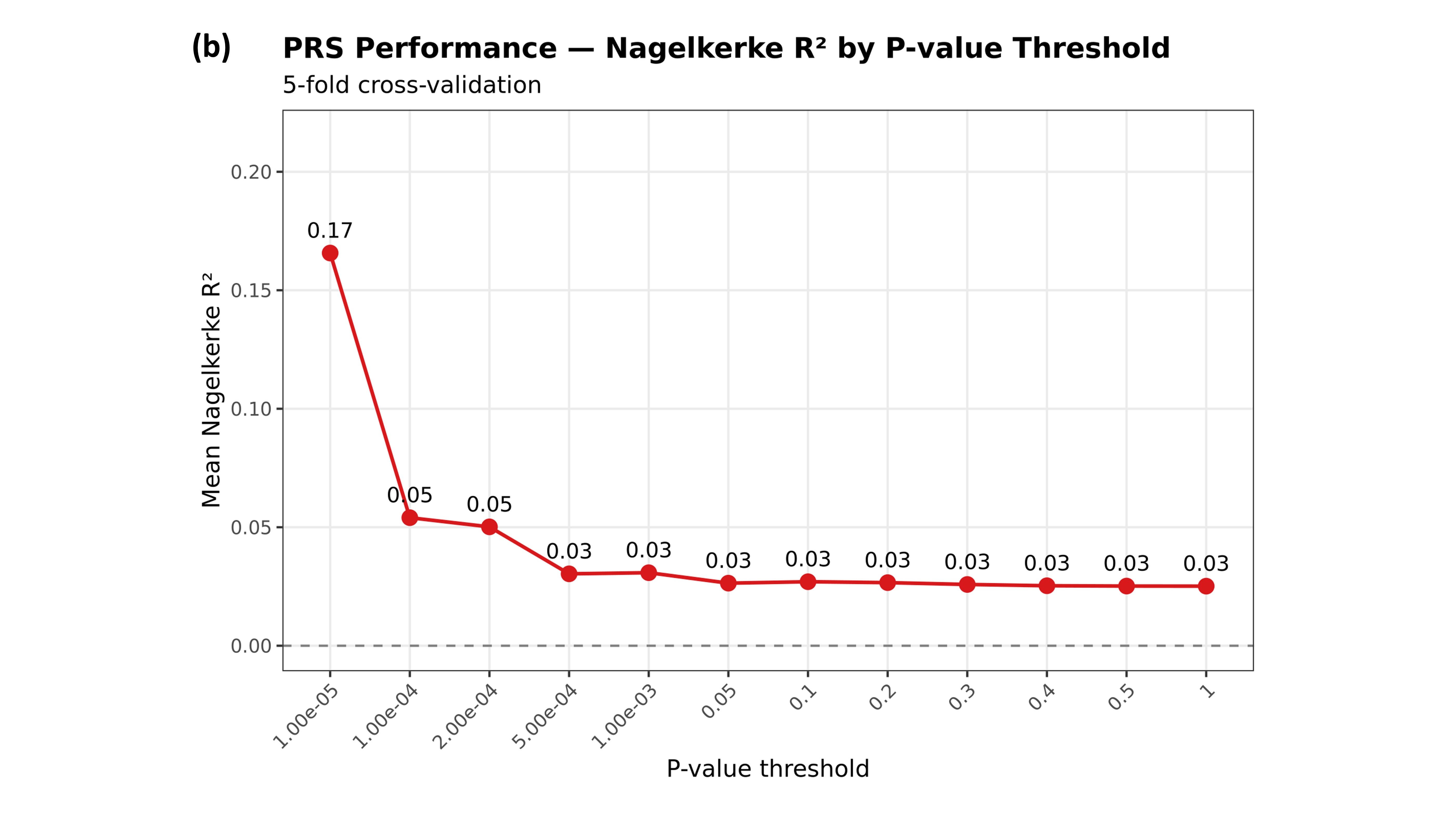

Supplement: Supplementary file 1 [file ijms-27-05165-s001.zip › Supplementary Figure S2b.tif]

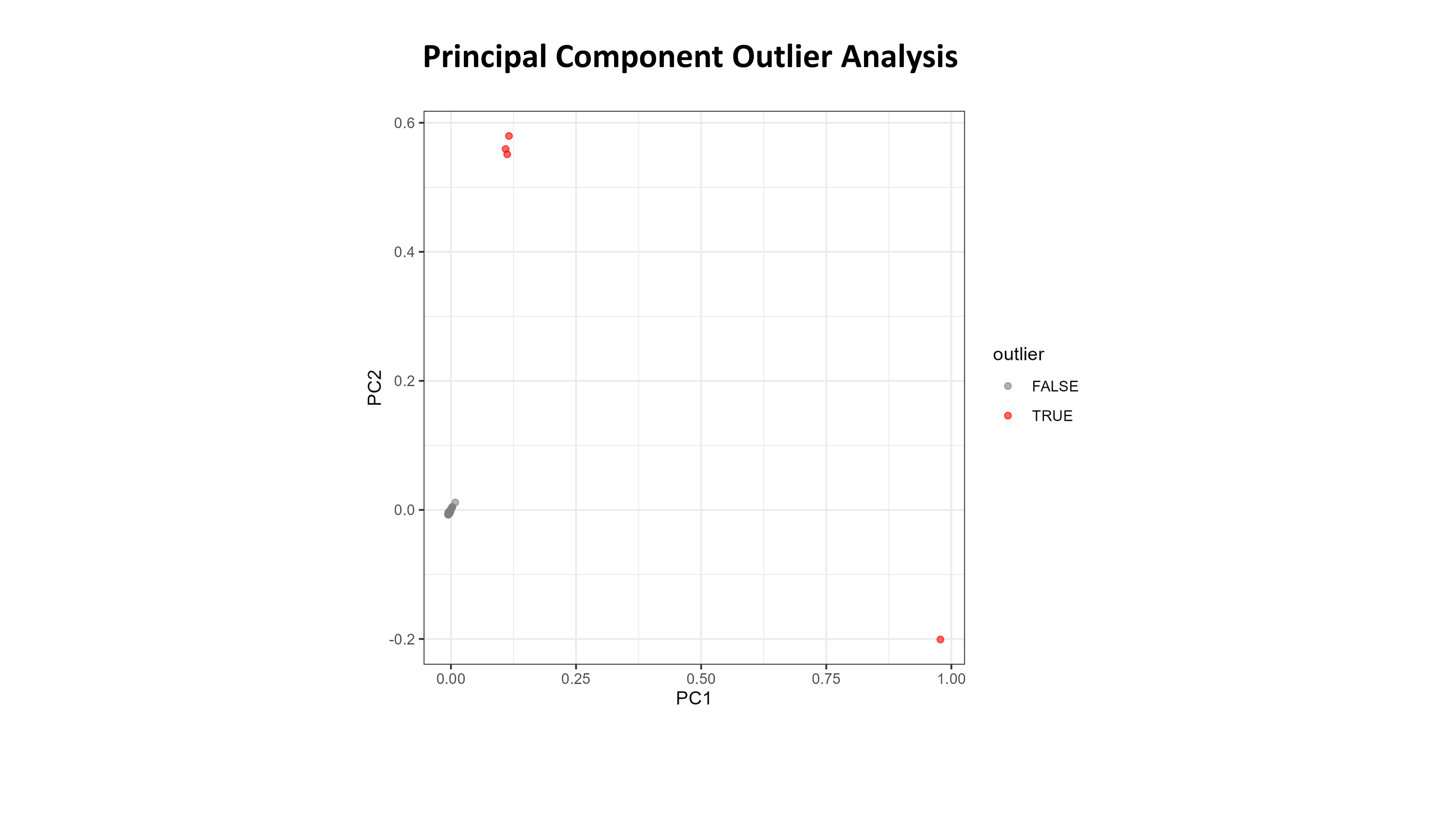

Supplement: Supplementary file 1 [file ijms-27-05165-s001.zip › Supplementary Figure S3.tif]
